# Neurogenic Hypertension Mediated Mitochondrial Abnormality Leads to Cardiomyopathy: Contribution of UPR^mt^ and Norepinephrine-miR- 18a-5p-HIF-1α Axis

**DOI:** 10.3389/fphys.2021.718982

**Published:** 2021-11-29

**Authors:** Shyam S. Nandi, Kenichi Katsurada, Sushil K. Mahata, Kaushik P. Patel

**Affiliations:** ^1^Department of Cellular and Integrative Physiology, University of Nebraska Medical Center, Omaha, NE, United States; ^2^Metabolic Physiology and Ultrastructural Biology Laboratory, Department of Medicine, University of California, San Diego, San Diego, CA, United States; ^3^VA San Diego Healthcare System, San Diego, CA, United States

**Keywords:** hypertension, mitochondrial damage, cardiac hypertrophy, cardiac fibrosis, HIF-1α, miR-18a-5p, UPR^mt^

## Abstract

**Aims:** Hypertension increases the risk of heart disease. Hallmark features of hypertensive heart disease is sympathoexcitation and cardiac mitochondrial abnormality. However, the molecular mechanisms for specifically neurally mediated mitochondrial abnormality and subsequent cardiac dysfunction are unclear. We hypothesized that enhanced sympatho-excitation to the heart elicits cardiac miR-18a-5p/HIF-1α and mitochondrial unfolded protein response (UPR^mt^) signaling that lead to mitochondrial abnormalities and consequent pathological cardiac remodeling.

**Methods and Results:** Using a model of neurogenic hypertension (NG-HTN), induced by intracerebroventricular (ICV) infusion of Ang II (NG-HTN; 20 ng/min, 14 days, 0.5 μl/h, or Saline; Control, 0.9%) through osmotic mini-pumps in Sprague-Dawley rats (250–300 g), we attempted to identify a link between sympathoexcitation (norepinephrine; NE), miRNA and HIF-1α signaling and UPR^mt^ to produce mitochondrial abnormalities resulting in cardiomyopathy. Cardiac remodeling, mitochondrial abnormality, and miRNA/HIF-1α signaling were assessed using histology, immunocytochemistry, electron microscopy, Western blotting or RT-qPCR. NG-HTN demonstrated increased sympatho-excitation with concomitant reduction in UPR^mt^, miRNA-18a-5p and increased level of HIF-1α in the heart. Our *in silico* analysis indicated that miR-18a-5p targets HIF-1α. Direct effects of NE on miRNA/HIF-1α signaling and mitochondrial abnormality examined using H9c2 rat cardiomyocytes showed NE reduces miR-18a-5p but increases HIF-1α. Electron microscopy revealed cardiac mitochondrial abnormality in NG-HTN, linked with hypertrophic cardiomyopathy and fibrosis. Mitochondrial unfolded protein response was decreased in NG-HTN indicating mitochondrial proteinopathy and proteotoxic stress, associated with increased mito-ROS and decreased mitochondrial membrane potential (ΔΨm), and oxidative phosphorylation. Further, there was reduced cardiac mitochondrial biogenesis and fusion, but increased mitochondrial fission, coupled with mitochondrial impaired TIM-TOM transport and UPR^mt^. Direct effects of NE on H9c2 rat cardiomyocytes also showed cardiomyocyte hypertrophy, increased mitochondrial ROS generation, and UPR^mt^ corroborating the *in vivo* data.

**Conclusion:** In conclusion, enhanced sympatho-excitation suppress miR-18a-5p/HIF-1α signaling and increased mitochondrial stress proteotoxicity, decreased UPR^mt^ leading to decreased mitochondrial dynamics/OXPHOS/ΔΨm and ROS generation. Taken together, these results suggest that ROS induced mitochondrial transition pore opening activates pro-hypertrophy/fibrosis/inflammatory factors that induce pathological cardiac hypertrophy and fibrosis commonly observed in NG-HTN.

## Introduction

Hypertension is an independent risk factor for heart failure, and is rapidly becoming a prominent threat, since it affects ∼80% of the elderly population (65 years and older) (Lionakis et al., 2012). Persistent sympatho-excitation and hypertension lead to cardiomyopathy and increases the risk of heart failure (Nadruz, 2015; Nandi et al., 2016). Hypertension causes heart failure in two ways; first, and most common, it induces left ventricular hypertrophy, fibrosis and stiffens the heart, which causes diastolic dysfunction (with preserved ejection fraction, HFpEF; Tam et al., 2017), and second, as a risk factor for myocardial infarction due to pressure overload (Volpe et al., 2016). Plasma levels of norepinephrine (NE) are known to be elevated during both hypertension and heart failure indicating an exaggerated sympatho-excitatory state (Patel et al., 2016). Blocking this exaggerated sympathetic drive with β-blockers has been useful to treat heart failure pathology. Further, attenuation of heart failure pathology with β-blockers has been attributed to relieving some of the mitochondrial dysfunction (Brown et al., 2017). Consistent with these observations, the β-blocker, carvedilol, has been clinically shown to be cardioprotective by improving mitochondrial metabolism and function (Igarashi et al., 2006). Metoprolol, another β-blocker, inhibits the defective accumulation of acyl-CoA and fatty acid uptake in the mitochondria (Nanki et al., 1987). However, the underlying molecular mechanisms linking sympatho-excitation and cardiac dysfunctions directly in hypertension remain unexplored (Eirin et al., 2018).

It has been reported that mitochondrial abnormalities are common feature in all types of cardiomyopathies (Shirakabe et al., 2016; Nandi et al., 2020a). However, the driving mechanisms underlying these mitochondrial abnormalities are largely unknown (Eirin et al., 2014). In all forms of heart disease, one common feature is abnormal mitochondrial structure due to either compromised mitochondrial metabolism/respiration, dynamics, quality control, reduced proteostasis, and altered import activities or impaired mitophagic clearance (Wrobel et al., 2015; Shirakabe et al., 2016). Newly discovered features of mitochondrial dysfunction associated with dynamics, proteostasis, and oxidative phosphorylation (OXPHOS) quality control factors in maintaining cardiac mitochondrial health are observed in clinical hypertension and cardiomyopathy. Furthermore, hypertrophic cardiomyopathy has been reported due to mitochondrial disease (Garcia-Diaz et al., 2013). Cardiomyopathy also has been reported in children and adults with mitochondrial disease (Holmgren et al., 2003; Sharp et al., 2014). Mitochondrial unfolded protein response (UPR^mt^) is a feature of retrograde mitochondrial signaling that influences to ensure the maintenance of mitochondria quality control mechanisms to stabilize the functional integrity of the mitochondrial proteostasis (Shpilka and Haynes, 2018; Rolland et al., 2019). When misfolded/misprocessed mitochondrial pre-proteins or unassembled mitochondrial supercomplex proteins accumulate beyond their folding capacity, it leads to altered protein trafficking of mitochondria, and induces mitochondrial proteotoxic stress and dysfunction (Wrobel et al., 2015). Under normal conditions, the heart relies on adequate mitochondrial ATP production to match myocardial energy demand, mostly through OXPHOS.

The discovery and understanding of miRNAs in regulation of cellular events have opened new therapeutic approaches for cardiovascular diseases (Nandi et al., 2015, 2016, 2018; Nandi and Mishra, 2018). Cardiac microRNAs that are downregulated during cardiac pathology appear to have a link to cardiomyopathy progression (Nandi and Mishra, 2015; Seeger and Boon, 2016). Expression of microRNAs is dynamically changed during cardiomyocyte hypertrophy, fibrosis, and cardiac remodeling. The miR-18a-5p is known as an anti-hypertrophy/anti-fibrosis miRNA in the heart (van Almen et al., 2011). Interestingly, cardiac miR-18a-5p is reduced in spontaneously hypertensive rats (SHR) (Huang et al., 2017), known to have increased sympathetic activation (Kline et al., 1983), suggesting a possible nexus between symptho-excitation and miR-18a-5p. Furthermore, miR-18a-5p is reduced in aged hearts and is associated with a decline in cardiac function (van Almen et al., 2011). This is thought to occur by regulating cardiac fibrosis by targeting pro-fibrotic miR-18a-5p genes in the heart. Moreover, loss of miR-18a-5p in the heart severely impairs cardiac function during hypertension, and restoration of cardiac-specific miR-18a-5p expression in SHR alleviated the cardiac dysfunction (Huang et al., 2017). We have reported that the “miRNA mimic” treatment mitigates cardiac fibrosis and hypertrophy in diabetic hearts (Nandi et al., 2016, 2018). Interestingly, previous studies have also demonstrated a role for miR-18a-5p in optimizing HIF-1α gene and protein expression during inflammation, cell survival, proliferation, and migration (Montoya et al., 2017). Further, HIF-1α mRNA levels have been shown to be regulated in an oxygen-independent manner by neurohumoral activators such as NE (Nikami et al., 2005), commonly elevated in hypertension (Zucker et al., 2001). However, the contribution of enhanced NE mediated changes in miR-18a-5p possibly *via* HIF-1α axis in the regulation of mitochondrial abnormalities within the hearts remains to be examined. Increased HIF-1α protein has been found in heart samples from patients with heart disease (Hölscher et al., 2012). The long-term stabilization and persistent activation of HIF-1α promote cardiac hypertrophy in hypertension and pressure overload heart disease (Krishnan et al., 2009; Hölscher et al., 2012; Fukai et al., 2015; Kumar et al., 2018). Furthermore, it has been shown that increased HIF-1α suppresses cardiac mitochondrial function (Kim et al., 2006; Papandreou et al., 2006; Kumar et al., 2018). Despite this information, the mechanisms of mitochondrial dysfunction and metabolic maladaptation are largely unknown in the heart during hypertension.

The aim of study is designed to explore the mechanistic link between exaggerated sympathetic drive, miRNA/HIF-1α signaling and cardiac mitochondrial abnormalities and associated development of pathological cardiac remodeling in a rat model of neurogenic hypertension (NG-HTN).

## Materials and Methods

### Animals and Ethics

Male Sprague-Dawley rats (250–300 g) from the Jackson Laboratory were housed in the animal facility of the University of Nebraska Medical Center. Rats were kept in hygienic cages in a room maintained at 22–24°C temperature, 30–40% humidity, with a 12 h of dark-light cycle, and provided *ad libitum* food and water. All experimental protocols were approved by the Institutional Animal Care and Use Committee, University of Nebraska Medical Center and all protocols/methods were conducted in accordance with the relevant guidelines and regulations of our institutional, the American Physiological Society, and the National Institutes of Health Guide for the Care and Use of Laboratory Animals.

### Neurogenic Hypertension Model

We used healthy male Sprague-Dawley rats aged 12 weeks, weighing 250–300 g and subjected them to intracerebroventricular (ICV) Ang II to develop the rat model of NG-HTN. Rats were randomly assigned to two groups, Saline ICV (Control) and Ang II ICV (NG-HTN). For ICV brain cannulation and mini-pump survival surgery, rats were anesthetized using a single i.p. injection of Ketamine (87 mg/kg) and Xylazine (10 mg/kg) mixture. A longitudinal skin incision was made on the head to expose the bregma and a small burr hole was made in the skull to access the dura. Rats were placed in a stereotaxic apparatus for infusion into the ICV space by stereotaxic implantation of an Alzet cannula of Brain Infusion Kit1 into the lateral ventricle. The tip of the cannula implanted was 1.5 mm lateral to the midline, 4.0 mm ventral to the dorsal surface and 0.8 mm caudal to bregma, as per Paxinos and Watson atlas (Paxinos and Watson, 2005). The cannula was connected to an osmotic minipump (Alzet, model 2002) for ICV infusion of Ang II (20 ng/min) or sterile isotonic saline as vehicle control for 14 days. Ang II infused rats that lacked dipsogenic response were considered not to have viable Ang II ICV infusion and therefore were excluded from the study.

### *In vivo* Hemodynamic Measurements

At the end of 14 days of ICV treatment, hemodynamic parameters were recorded using a Mikro-Tip catheter (SPR-407), Millar Instruments; Houston, TX, United States) with a non-survival terminal procedure in an experimenter-blinded fashion. In brief, animals were anesthetized using a single injection of urethane (0.75 g/kg i.p.) and chloralose (60 mg/kg i.p.). The catheter was inserted into the LV chamber *via* the right carotid artery as described previously (Nandi et al., 2016). The mean arterial pressure (MAP), heart rate (HR), and hemodynamic parameters were simultaneously recorded on a PowerLab data acquisition system (8SP, ADInstruments, United States) and analyzed as reported previously (Nandi et al., 2016; Patel et al., 2016). The cardiac responsiveness (change in ±dP/dt) were measured with intravenous infusion of 0.5 μg/kg isoproterenol (a β-AR agonist). At the end of the experiment, rats were euthanized by Fatal Plus euthanasia solution (120 mg/kg pentobarbital, i.p.).

### Assessment of Cardiac Function by M-Mode Echocardiography

Rats were lightly anesthetized with 1.5% isoflurane nose cone. M-mode echocardiography (using Vevo 3100 Imaging System, VisualSonics) was performed at 5 days of ICV post-surgery recovery (for baseline) and at the end of 14 days of ICV saline or Ang II mini-pump infusion. Left ventricular mitral filing parameters (E/A) was measured and compared with respective baseline for the assessment of diastolic dysfunction in NG-HTN rats.

### Western Immunoblotting

The routine Western immunoblotting protocol was used for protein expression analysis. Cell or tissue protein lysates was estimated by protein assay (BCA Kit, cat# 23227, Pierce, United States). Boiled protein lysate (25 μg/lane) in 4× denaturing Laemmli Sample Buffer (cat# 161-0747, Bio-Rad Laboratories, United States) were loaded on SDS-PAGE. Next, gels were transferred onto a PVDF (Polyvinylidene fluoride) membrane. Transferred membranes were incubated with 5% milk (Bio-Rad, non-fat dried milk) in TBS for 60 min at room temperature and washed twice in TBS for 5 min each. Blots were then incubated overnight at 4°C with diluted primary antibodies in TBS (1:1000). The primary antibodies used were OXPHOS WB antibody cocktail assay (cat# ab110413), FIS1 (cat# ab71498), OPA1 (cat# ab157457), TIM17 (cat# ab192246), TOM20 (cat# ab186734), HIF-1α (cat# ab179483), ATF5 (cat# ab184923), and VDAC1/Porin (cat# ab14734) was from Abcam, and HSP60 monoclonal antibody (cat# ADI-SPA-806-D, Enzo Life Science), YME1L1 (cat# 11510-1-AP, Proteintech), and Mn-SOD (cat# 06-984) was from Millipore Sigma. The β-actin antibody (cat# Sc-47778) was from Santa Cruz Biotechnology, United States. Respective secondary antibodies with HRP conjugates were diluted at 1:4000 in TBS and incubated at room temperature for 2 h. After washing in TBST (3 × 5 min) membranes were developed using SuperSignal^®^ West Femto Stable peroxidase buffer (cat# 1859023, Thermo Scientific, United States) using a ChemiDoc™ XRS Molecular Imager (Bio-Rad Laboratories, United States). Restorer-restriping buffer (cat# 46430) was used for membrane stripping. The images were captured using the Image Lab software version 6 (Bio-Rad Laboratories, United States).

### Isolation of Mitochondrial Proteins

Freshly isolated left-ventricle tissue of hearts were processed for the separation of cytosolic and mitochondrial fractions using pierce mitochondria isolation kit (cat# 89801) as per manufacturer’s instructions. In brief, tissues were lysed and homogenized in series of extraction buffers and proceeds for mitochondrial and cytosolic protein isolation by centrifugations. CHAPS buffer (2% CHAPS in Tris buffered saline) was used to lyse the mitochondrial pellets at 4°C. Isolated proteins were quantified using standard BCA protein assay kit (cat# 23225) and processed for Western blot measurements.

### Wheat Germ Agglutinin Staining

Wheat germ agglutinin (WGA) staining was performed on 5 μm transverse cryosections of the heart. Heart cryosections were prepared using a CryoStarNX50 (Thermo Fisher Scientific, United States) and fixed in 4% paraformaldehyde solution for 30 min. Sections were washed 2 × 5 min in TBS post-fixation and then incubated with WGA staining solution (5 mg/ml, cat# W834, Thermo Fisher Scientific, United States) for 15 min at RT. Next, sections were washed and mounted with coverslip, and images were captured using a fluorescence microscope (Olympus IX71 Imaging Systems) to measured mean cardiomyocyte diameter and numbers per unit area.

### Masson’s Trichrome Staining

To determine cardiac fibrosis, we performed Masson’s trichrome staining, where the blue color staining represents collagen fibers. We used Masson’s Trichrome kit (cat# 87019, Thermo Fisher Scientific, United States) to stain 5 μm paraffin transverse sections of the heart. We calculated perivascular (PV) and interstitial (INT) fibrosis by quantifying % blue pixel intensity/total pixel intensity of areas using color deconvolution tool of Fiji ImageJ software, NIH. We used the Tissue Core Facility of the University of Nebraska Medical Center for Masson’s Trichrome staining procedure.

### Picrosirius Red Staining

To corroborate Masson’s trichrome staining results, we performed Picrosirius red staining. In brief, 10% formalin-fixed left ventricular paraffin sections (5 μm) were processed for picrosirius red staining. The reagents were Direct Red 80 (cat# 365548) and picric acid (cat# P6744-1GA) from Sigma Aldrich, United States, and glacial acetic acid (cat# A38-50) from Thermo Fisher Scientific, United States. We followed the standard kit protocol for this staining at the Tissue Core Facility of the University of Nebraska Medical Center.

### Transmission Electron Microscopy

The left ventricular heart samples were processed for evaluation by transmission electron microscopy (TEM) as described previously (Nandi et al., 2020a). Briefly, 2 mm thin slices are immersion fixed with 2.5% glutaraldehyde and 2% paraformaldehyde in 0.15 M cacodylate buffer and post-fixed in 1% OsO4 in 0.1 M cacodylate buffer for 1 h on ice, followed by en bloc staining with 2–3% uranyl acetate for 1 h on ice. Next, the tissue slices are dehydrated using series of graded ethanol (20–100%) on ice followed by a single wash with 100% ethanol and subsequent two washes with acetone (15 min each) and finally embedded with Durcupan. Leica UCT ultramicrotome was used for ultrathin tissue sections (50–60 nm), which were adhered to Formvar and carbon-coated copper grids. Grids were stained with 2% uranyl acetate for 5 min and Sato’s lead stain for 60 s. A JEOL JEM1400-plus Transmission Electron Microscope (JEOL, Peabody, MA, United States) was used to view the grids and a Gatan OneView digital camera with 4000 × 4000 resolution (Gatan, Pleasanton, CA, United States) was used to take the photographs. NIH ImageJ tool was used to calculate the area by manual tracing around the mitochondria and cristae. Mitochondrial area was calculated by dividing the total mitochondria area with the area of the cell and multiplied by 100. Likewise, cristae area was determined by dividing the total cristae area with the total area of mitochondria and multiplied by 100 as described previously, and the number of autophagosomes was also calculated as per our previous publication. All these quantifications were done with a blinded fashion.

### Mito-SOX Staining of Heart Sections

Mitochondrial superoxide assay was performed using Mito-SOX Red (cat# M36008, Life Technologies, United States) staining, Mito-SOX is a fluorogenic dye that specifically stains mitochondrial superoxide in live cells. Oxidation of Mito-SOX produces red fluorescence upon binding to mitochondrial nucleic acid. H9c2 cells were incubated with 5 μM of Mito-SOX in incomplete medium and cells were incubated for 10 min at 37°C, followed by a quick wash with PBS, and images were captured immediately under a fluorescence microscope (Olympus IX71 Imaging Systems). The Mito-SOX intensity was quantified by ImageJ software (NIH, United States).

### Immunohistology

Immunofluorescence (IF) histology was performed on 5 μm transverse cryosections. Standard protocol was used for these staining as per our previous publications (Nandi et al., 2016). The primary (1:100) and secondary antibodies (1:200) used were; HIF-1α (cat# ab179483), and Sarcomeric alpha-actinin (cat# ab9465) from Abcam, anti-mouse Alexa^®^Fluor 594 (cat# A21201) and anti-rabbit Alexa^®^Fluor 488 (cat# A21441) from Life Technologies, and anti-rabbit-HRP (cat# sc-2054) from Santa-Cruz Biotechnology. Images were captured using bright-field microscope (Leica Microsystems, United States) and Olympus IX71 fluorescence Imaging Systems (Olympus, United States) respectively. Images were quantified using ImageJ software (NIH, United States).

### Immunocytochemistry

Immunocytochemistry was performed on *in vitro* cultured rat H9c2 cardiomyocytes. In brief, cardiomyocytes were washed in PBS and fixed with 4% paraformaldehyde solution for 40 min. Cells were permeabilized with 0.025% Triton-X-100 (v/v) solution in PBS for 40 min and blocked in 1% sterile BSA solution for 60 min at RT. After blocking, cells were washed and incubated in 1:100 diluted primary antibody anti-ANP (cat# GTX109255, GeneTex, United States) in PBS at 4°C for overnight. On the next day, cells were washed in PBS and incubated with 1:200 dilution of anti-rabbit Alexa^®^Fluor 488 conjugated secondary antibody (cat# A11008, Life Technologies, United States). Cardiomyocyte nuclei were counterstained with 1 μg/ml DAPI in PBS (cat #A1001, AppliChem, United States) and Alexa^®^Fluor 594 Phalloidin (cat# A12381, Life Technologies, United States) was used to counterstain the F-actin filaments. Fluorescence images were captured by Olympus IX71 Imaging Systems (Olympus, United States) and quantified with ImageJ software (NIH, United States).

### F-Actin Staining of Cardiomyocytes

Rat H9c2 cardiomyocytes were fixed with 4% paraformaldehyde for 40 min at RT, followed by permeabilization with 0.5% Triton-X-100 (v/v) in PBS for 40 min. After fixation, cells were incubated with Alexa Fluor^®^594 Phalloidin, 200 μM (cat# A12381, Life Technologies, United States) in PBS for 40 min and protected from direct light, and then washed with PBST. Images were captured by Olympus IX71 Imaging Systems (Olympus, United States) and the F-actin red intensity was quantified by ImageJ software (NIH, United States).

### TMRE Assay

In order to evaluate mitochondrial membrane potential (ΔΨm), cultured rat H9c2 cardiomyocytes are stained with TMRE dye (cat# T669, Life Technologies) as per the manufacturer’s instruction. TMRE is a cell-permeant, cationic, red-orange fluorescent dye that is readily sequestered by active mitochondria. H9c2 cardiomyocytes are incubated with TMRE dye (50 nM) for 40 min at 37°C in incomplete DMEM medium following the manufacturer’s instructions. Post-staining the TMRE-incubated medium is replaced with fresh incomplete DMEM medium, and fluorescent microscopic images are captured using a fluorescence microscope (Olympus IX71 Imaging Systems). TMRE intensity was quantified by ImageJ software (NIH, United States).

### MiR-18a-5p Assay

Standard miRNA assay protocols were used for the quantification of miR-18a-5p levels (Nandi et al., 2016). We used total RNA extraction and TaqMan based primers for miRNA assays. In brief, the mirVana miRNA Isolation Kit (cat# AM1560) was used to extract total RNAs from heart and cultured cardiomyocytes. The highly pure quality of RNA (A260/A280 > 1.8–2.0) was quality checked by a NanoDrop 2000c (Thermo Fisher Scientific, United States). TaqMan miRNA reverse transcription kit (cat# 4366597, Life Technologies, United States) was used for the first strand synthesis, and then in the second step PCR reaction mixture was amplified using TaqMan based primers specific for miR-18a-5p (Assay ID:002422, Life Technologies, United States). TaqMan U6 SnRNA (assay ID: 001973, Life Technologies, United States) was used as an endogenous control for the miR-18a-5p. Bio-Rad CFX qPCR instrument (Bio-Rad Laboratories, United States) was used to run the PCR reaction mixtures and data were analyzed by a Bio-Rad CFX Manager software (Bio-Rad Laboratories, United States).

### Luciferase Reporter Assay

We performed a miRNA target validation luciferase reporter assay using H9c2 cells. In brief, cells were transfected with lentiviruses either GFP-tagged miR-18a-5p (cat# RmiR6078) or scrambled miRNA (cat# CmiR0001), which were purchased from GeneCopoeia. We received custom-designed HIF-1α 3′ untranslated region (UTR) clones (WT 3′UTR: cat# RmiT050798; mutant 3′UTR: cat# CS-RmiT050798) from GeneCopoeia, Rockville, MD, United States. For luciferase reporter assay, we co-treated 1 μg of WT or mutant HIF-1α 3′UTR clones with either miR-18a-5p or GFP-scrambled overexpression for 48 h, and measured the relative firefly to Renilla luciferase activity using a Dual-Glow Luciferase Assay kit (cat# E2920, Promega Corp., Madison, WI, United States) and a GloMax-Multi + Detection System (Promega) following the manufacturer’s manual.

### MiRNA–mRNA Electrophoretic Mobility Shift Assay

We followed the published protocol for the miRNA–mRNA EMSA (Nandi and Mishra, 2017). For binding assay oligonucleotides were custom synthesized (IDT, United States). The sequences used are listed in Table 2. Probes were incubated in EMSA binding buffer (10 mM MgCl_2_, 100 mM NaCl, 50 mM HEPES pH 7.2 and 5% glycerol) for 30 min at 37°C with corresponding WT or mutant 3′UTR oligonucleotides. Binding reactions were electrophoresed in a 12% PAGE (10 mM MgCl_2_, 50 mM HEPES pH 7.2 and 5% glycerol) for 2 h at 190V at 4°C. EMSA was performed using fluorescence-based EMSA Kit (cat# E33075, Thermo Scientific Inc., United States) and labeling of the oligonucleotides was performed with SYBR green EMSA nucleic acid gel stain. The SYBR Green stained gel was scanned in a Chemidoc (ChemiDoc, Image Lab 4.1, Bio-Rad Laboratories, United States), using a SYBR Green filter with UV trans-illumination.

### H9c2 Cardiomyoblast Culture, Differentiation and *in vitro* Treatment

The H9c2 rat myofibroblasts were cultured following the standard protocol ATCC^®^. Cells were cultured in DMEM high glucose (cat# D5796, Sigma) with 10% FBS (Sigma, F6178) and supplemented with 1% penicillin/streptomycin in a CO_2_ incubator (Thermoelectric Corporation, 800-WJ, United States) at 37°C with 5% CO_2_. H9c2 myofibroblast were allowed to differentiate toward cardiomyocytes in presence of 1 μM retinoic acid (RA) and 1%FBS (cat# F2442, United States) for 7 days. Differentiated H9c2 were subjected to norepinephrine (2 μM) treatment for 48 h and post-treatment cell samples were trypsinized to isolate RNA and proteins using standard protocols. MiR-18a-5p mimic (cat# 4464066), inhibitor (cat# 4464084), and scrambled negative control (cat# 4464058) used for miRNA gain and loss of function assays were from Life Technologies. The pDsRed2-Mito Vector (cat# 632421) was from Clontech.

### Statistical Analysis

Data are presented as mean ± SE. Means ± SE between two groups were compared with a Student’s *t*-test. Differences between more than two groups were determined by one- or two-way ANOVA, followed by the Tukey’s multiple comparisons test for *post hoc* analysis if there was a significant interaction using Prism7.0 GraphPad software. *P*-value < 0.05 is considered statistically significant.

## Results

### Neurogenic Hypertension, Cardiac Hypertrophy, and Fibrosis

**FIGURE 1:**
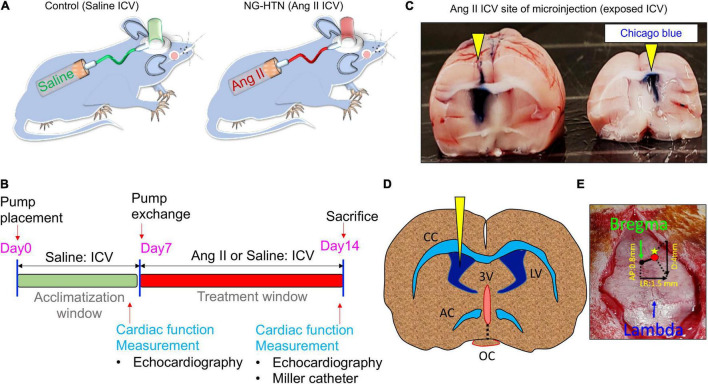
Schematic showing chronic Ang II intracerebroventricular (ICV) infusion timeline and protocol to develop NG-HTN. **(A)** Schematic showing development of rat model of neurogenic hypertension *via* ICV Ang II brain minipump infusion. Sprague-Dawley rats (250–300 g) subjected to ICV Ang II brain minipump infusion (20 ng/min, 14 days, 0.5 μl/h) or vehicle (control, 0.9% sterile saline infusion) through osmotic mini-pumps. **(B)** Schematic timeline of treatment and cardiac function measurements. **(C)** Representative whole brain image showing exposed ICV cannulation site of Ang II or saline infusion confirmed by Chicago blue dye flushing through the cannulation tube at the time of sacrifice (yellow arrowheads). **(D)** Schematic coronal brain slice shown for ICV cannulation. **(E)** Stereotactic co-ordinate measurements of brain cannulation and ICV pump implantation, AP, LR, and D co-ordinate measurement are shown with a referenced from the bregma.

The consequence of ICV infusion of Ang II was an increase in MAP compared to the Saline (ICV) infused Control group (125 ± 8 mmHg; Ang II vs. 83 ± 4 mmHg; Saline) with a concomitant increase in cardiac sympathetic tone (95 ± 6 bpm; Ang II vs. 73 ± 6 bpm Saline) as well as basal renal sympathetic nerve activity (19.8 ± 2.0%, Ang II vs. 6.9 ± 1.9%; Saline of Max). In addition, the left ventricular end diastolic pressure (LVEDP) increased slightly in NG-HTN (3.6 ± 0.5 mmHg; NG-HTN vs. 2.0 ± 0.3 mmHg; Control) but not significantly. There was an increase in HW/BW(g) ratio (4.5 ± 0.4; NG-HTN vs. 2.8 ± 0.06; Control). Echocardiography measurements showed decreased mitral filing velocity (E/A ratio, NG-HTN: 1.2 ± 0.1 vs. 1.5 ± 0.2; Control), which confirmed that rats with NG-HTN developed diastolic heart failure. Furthermore, cardiac contractile responsiveness to isoproterenol (0.5 μg/kg ISO) was significantly lower for negative dP/dt in NG-HTN rats (NG-HTN: −8581 ± 661 vs. −11,765 ± 708; Control) which is indicative of abnormal diastole or relaxation in NG-HTN rats. Additional characteristics and experimental timeline details of Control and NG-HTN rats are provided in the Figures 1A–E and Table 1.

**TABLE 1:** Characteristics of Control and NG-HTN rats used in the studies.

Parameters	Control (*n* = 6)	NG-HTN (*n* = 6)
Body weight; BW (g)	331.1 ± 11.2	235.3 ± 18.2*
Heart weight; HW (g)	0.94 ± 0.02	1.03 ± 0.03*
HW/BW × 1000	2.86 ± 0.06	4.54 ± 0.45*
Heart rate; HR (bpm)	372 ± 13	386 ± 11
+dP/dt (mmHg/s)	7258 ± 486	7778 ± 404
−dP/dt (mmHg/s)	7987 ± 860	8387 ± 561
MAP (mmHG)	83 ± 4	125 ± 8
LVEDP (mmHg)	2.0 ± 0.3	3.6 ± 0.5
E/A	1.5 ± 0.2	1.2 ± 0.1*
%EF	69 ± 5%	67 ± 2%
−dP/dt with ISO	−11,765 ± 708	−8581 ± 661*

LVEDP, left ventricular end-diastolic pressure; MAP, mean arterial pressure; HR, heart rate; EF, ejection fraction; ISO, Isoproterenol. Data are represented as mean ± SE of six rats in each group. *P < 0.05 sham vs. NG-HTN. Student’s t-test.

**FIGURE 2:**
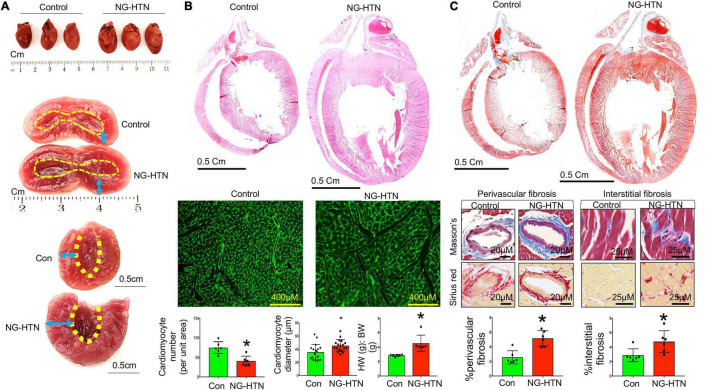
Neurogenic hypertension induces cardiac hypertrophy and fibrosis. **(A)** Top, whole heart images of control and NG-HTN hearts, *n* = 3. Middle, representative image showing exposed hearts chambers. Bottom, endocardium outlined with dotted line, arrow space highlights wall thickness. **(B)** Top, HE stained images showing longitudinal whole heart sections of control and NG-HTN rats. Middle, WGA-488 staining of heart sections, green represents cardiomyocyte boundaries stained with WGA. Bottom, quantifications of cardiomyocyte numbers (each dot represent number of cardiomyocytes count per image, *n* = 6 rats) and cardiomyocyte diameter (μm, per unit area, each dot represent individual cardiomyocytes diameter per section from *n* = 6 rats), and heart weight (HW) to body weight (BW) ratio in control vs. NG-HTN rats, *n* = 6 rats. **(C)** Top, representative Masson’s trichrome (MT) stained longitudinal whole heart sections, blue represents collagen staining. Middle, representative MT (blue), and Picrosirius (red) images of whole heart sections showing perivascular and interstitial fibrosis. Bottom, quantification for % fibrosis in control and NG-HTN rats, each dot represents Mansson’s blue intensity per unit area. Values are presented as mean ± SEM, *n* = 6. *P < 0.05 vs. Control, Student’s *t*-test.

To characterize and identify features of cardiac remodeling in NG-HTN we measured morphometry, hypertrophy, and indices of fibrosis in the heart. First, to investigate whether NG-HTN cause a change in the gross cardiac shape and size we monitored heart morphometry along with the left ventricular anatomy (Figure 2A). The results showed that NG-HTN increased LV wall-thickness. Next, we analyzed heart weight to body weight ratio (HW:BW), which showed an increase in the NG-HTN group compared to Control group, suggesting cardiac hypertrophy in rats with NG-HTN (Figure 2B). To further validate cardiac hypertrophy, we performed H&E staining of the longitudinal whole heart sections in order to visualize the atrial and ventricular wall histology. Our results confirmed an increase in gross heart size and thickening of the left ventricular wall in NG-HTN rats (Figure 2B, top panel). WGA-Fluorescein (Wheat Germ Agglutinin-Fluorescein) staining of the left ventricular transverse sections (Figure 2B, bottom panel) determined the hypertrophic changes at the level of the cardiomyocyte. WGA is a lectin, which specifically stains the sialic acid and N-acetylglucosaminyl residues delineating cardiomyocyte boundaries allowing the quantification of cardiomyocyte number and diameter per unit area of left ventricular tissue. NG-HTN increased cardiomyocyte size and decreased the number of cardiomyocytes per unit area of left ventricular section (Figure 2B) suggesting pathological hypertrophic changes at the level of cardiomyocytes. Masson’s trichrome staining on transverse and longitudinal cardiac sections (Figure 2C, top and middle panel) determined widespread cardiac fibrosis (blue). This was corroborated by similar increase in perivascular, and interstitial collagen deposition (red) in picrosirius red stained hearts NG-HTN group (Figure 2C, bottom panel). Our data demonstrate an increase in perivascular and interstitial collagen deposition in NG-HTN rats (Figure 2C), indicating an increase in cardiac fibrosis. Taken together, the cardiac pathology associated with our model of NG-HTN showed hypertrophic cardiomyopathy that is similar to cardiomyopathy complications commonly observed in patients with hypertension.

### Neurogenic Hypertension and Cardiac Mitochondrial Dysfunction

#### Mitochondrial Abnormality

**FIGURE 3:**
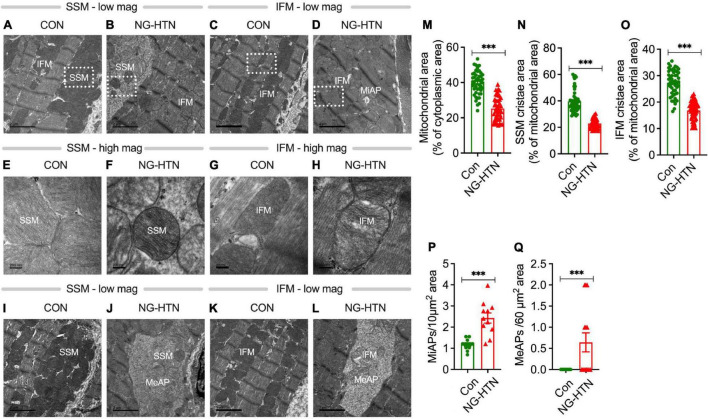
Neurogenic hypertension and cardiac mitochondrial injury. **(A–D)** Low magnification EM micrographs showing mitochondrial population and microautophagosomes. **(E–H)** Higher magnification of a selected area in **(A–D)** showing the mitochondrial ultrastructure abnormalities. **(I–L)** Low magnification EM micrographs showing macroautophagosomes. Morphometric analyses: **(M)** mitochondrial area, **(N)** SSM cristae area, **(O)** IFM cristae area, **(P)**, microautophagosomes, and **(Q)** macroautophagosomes. Mitochondrial area was calculated from 15 micrographs per rat heart (dot represents 45 individual mitochondria counts), *n* = 3 rats. Cristae area was calculated from at least 15 micrographs from each rat (dot represents 50 individual mitochondria counts), *n* = 3 rats. Microautophagosomes were calculated from 11 micrographs representing *n* = 3 rats, dot represents numbers per unit area. Megaautophagosomes were calculated from 14 micrographs representing 3 rats, dot represents numbers/unit area. ***P < 0.001 vs. Control, Student’s *t*-test. Scales bars: **(A–D)**, 2 nM; **(E–H)**, 200 nM; and **(I–L)**, 2 nM.

Alterations in mitochondrial ultrastructure, at the level of the cristae, were examined by electron microscopy (EM) on left ventricular tissues from Control and NG-HTN rats. NG-HTN resulted in marked decrease in mitochondrial area (by 36%) as well as decrease in cristae area in subsarcolemmal (SSM: by 42%) and inter/intra-myofibrillar mitochondria (IFM: by 38%) (Figures 3A–H,M–O), indicating a reduced in mitochondrial quality control mechanisms and function. While increased numbers of microautophagosomes (MiAPs) were detected in NG-HTN hearts compared to WT hearts, macroautophagosomes (MeAPs) were exclusively observed in the NG-HTN hearts (Figures 3I–L,P,Q) implicating profound changes caused by NG-HTN. Marked decrease in mitochondrial abundance coupled with significant decrease in cristae surface area indicate compromised heart function in NG-HTN group.

### Mitochondrial ROS Generation, Depolarization, and Altered Oxidative Phosphorylation

**FIGURE 4:**
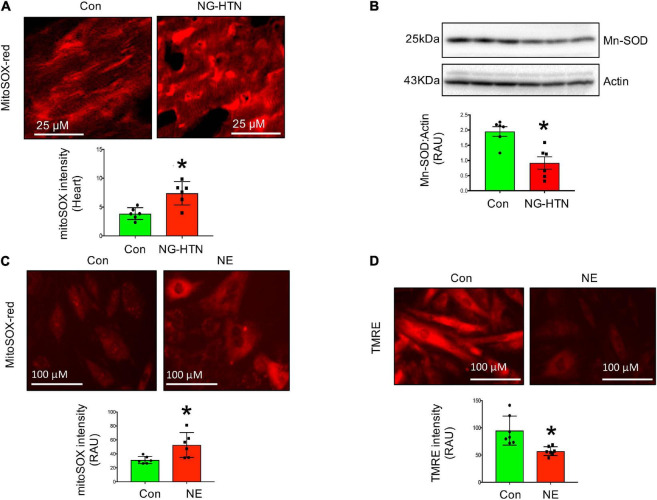
Neurogenic hypertension and cardiac mitochondrial quality control events. **(A)** Top, Mito-SOX red (mitochondrial ROS marker) staining of control and NG-HTN heart sections. Bottom, quantification of Mito-SOX intensity. **(B)** Western blot to assess Mn-SOD protein level in control and NG-HTN whole hearts lysate, bottom, WB quantification of Mn-SOD. **(C)** Live cell Mito-SOX imaging and quantification showing Mito-SOX intensity in control and NE-treated H9c2 cardiomyocytes. **(D)** Live cell TMRE imaging and quantification showing mitochondrial membrane potential (ΔΨm) in control and NE-treated H9c2 cardiomyocytes. Values are mean ± SEM, *n* = 6 rats, *P < 0.05 vs. Control, Student’s *t*-test.

**FIGURE 5:**
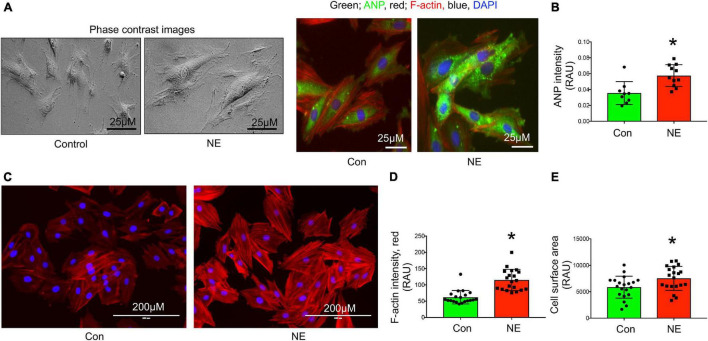
Norepinephrine induced hypertrophy in H9c2 rat cardiomyocytes. **(A)** Representative phase contrast and immunocytochemistry images showing cardiomyocyte hypertrophy marker ANP localization in control and NE-treated H9c2 cardiomyocytes. **(B)** Bargraph showing quantification of ANP intensity (green), *n* = 10 cells per group, dot represent individual cell intensity from three independent experimental groups. **(C)** Representative images showing F-actin staining using Alexa^®^Fluor 594 Phalloidin conjugate dye of control and NE-treated H9c2 cardiomyocytes. Red, F-actin; blue, DAPI stained nucleus. **(D)** Bargraph showing quantification of F-actin intensity and **(E)** cell surface area measurement in control and NE-treated H9c2 cardiomyocytes, *n* = 20 cells per group, dot represent individual cells intensity or area from three independent experimental groups. Data are presented as mean ± SEM. *P-values < 0.05 considered as significant, Student’s *t*-test.

Mitochondrial superoxide staining of cardiac tissue sections using mitochondrial superoxide-specific dye Mito-SOX red, was performed to evaluate altered mitochondrial reactive oxygen species in hearts from rats with NG-HTN. There was an increase in Mito-SOX red intensity in the hearts of NG-HTN compared to Control rats (Figure 4A). To address the mechanism of increased superoxide we measured Mn-SOD protein level in control and NG-HTN heart lysate (Figure 4B). Our results showed that Mn-SOD protein level is decreased in the heart of NG-HTN. These results suggest a downregulated antioxidant defense mechanism resulting in an increased mitochondrial ROS generation. The direct effect of NE on mitochondrial ROS and mitochondrial transmembrane potential (ΔΨm) markers were evaluated by Mito-SOX staining assay in control and NE-treated H9c2 cardiomyocytes. NE caused an increase in Mito-SOX red intensity in isolated cardiomyocytes (Figure 4C), suggesting that depolarized ΔΨm induces mitochondrial superoxide production. Next, to assess mitochondrial depolarization, we performed TMRE (Tetramethylrhodamine, ethyl ester) assay in H9c2 cardiomyocytes. NE-treatment decreased mitochondrial ΔΨm, as indicated by a decrease in the TMRE red intensity in cardiomyocytes treated with NE (Figure 4D) and associated with cardiomyocyte hypertrophic changes (Figures 5A–E). These results suggest that NE-drive increased ROS, but decreased ΔΨm in NG-HTN.

**FIGURE 6:**
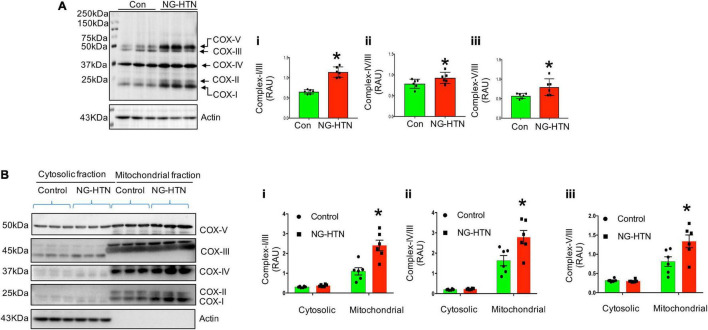
Neurogenic hypertension and cardiac mitochondrial defective OXPHOS subunit assembly/import. **(A)** Left, Western blot showed mitochondrial OXPHOS complex protein level in control and NG-HTN whole hearts lysate (total OXPHOS WB antibody cocktail, cat# ab110413, Abcam). Right, (i–iii) WB quantification of mitochondrial Complex-I/III, Complex-IV/III, and Complex-V/III ratio, respectively. **(B)** Left, Western blots showed mitochondrial OXPHOS complex protein level in cytosol vs. mitochondria fraction in control and NG-HTN hearts. Right, (i–iii) Western blot quantification of mitochondrial Complex-I/III, Complex-IV/III, and Complex-V/III ratio, respectively. Values are mean ± SEM, dot represents *n* = 6 rats, *P < 0.05 vs. Control, **(A)**, Student’s *t*-test, **(B)**; two-way ANOVA with Tukey’s multiple comparison test.

We further examined whether NG-HTN induces mitochondrial OXPHOS/respiratory dysfunction and altered expression of mitochondrial OXPHOS Complexes I, -II, -III, -IV, and -V, proteins. Although NG-HTN significantly increased Complex-I, II, IV, and V, Complex-III remained unchanged (Figure 6A). These results indicate an altered OXPHOS and a shifted mitochondrial metabolism in the hearts of rats with NG-HTN. This is possibly due to increased Complex-I expression that accepts reduced NADH carried energy to force protons into the mitochondrial intermembrane space for ATP synthesis *via* Complex-V. Our results demonstrate that OXPHOS supercomplex subunit abundance is increased in NG-HTN whole heart lysate, therefore we further compared the OXPHOS subunit level in cytosol vs. isolated mitochondria to distinguish a subunit assembly over an import defects. Our results confirmed that increased Complex-I and –IV, and -V abundance in mitochondrial compartments of NG-HTN hearts compared to control hearts (Figure 6B), while their cytosolic levels were comparable. These results support an OXPHOS subunit assembly defect perhaps associated with misfolded peptide accumulation due to reduced mitochondrial proteases activation.

### Mitochondrial Biogenesis and Fusion/Fission Dynamics

**FIGURE 7:**
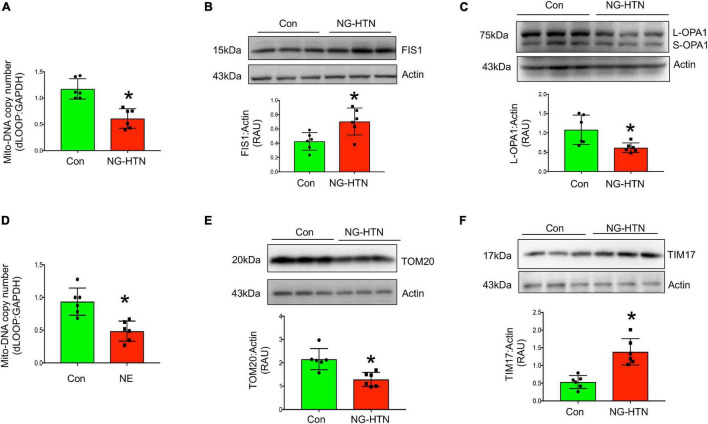
Neurogenic hypertension induces shifts in cardiac mitochondrial dynamics and protein transport. **(A)** Measurement of relative d-LOOP/GAPDH DNA ratio by qPCR from control and NG-HTN hearts. **(B,C)** Western blot representative and quantification showing expression of FIS1 and OPA1 level, receptively, normalized to Actin in control and NG-HTN hearts. **(D)** Measurement of relative d-LOOP/GAPDH DNA ratio by qPCR in control and NE-treated H9c2. **(E,F)** Representative Western blot and quantification showing expression of TOM20 and TIM17 level, respectively, normalized to Actin. Values are mean ± SEM, dot represents *n* = 6 rats, *P < 0.05 vs. Control, Student’s *t*-test.

Mitochondrial biogenesis, was assessed by measuring relative mt-DNA content compared to the nuclear DNA content. In addition, mitochondrial fission was evaluated by measuring the level of FIS1 that facilitates the fission of mitochondria. Levels of OPA1 that facilitates fusion of mitochondria, in combination with mitochondrial fragmentation and mitophagy vacuoles were assessed by EM (Figure 7C). Mitochondrial relative DNA content was significantly lower in the NG-HTN group suggesting decreased mitochondrial biogenesis (Figure 7A). Furthermore, mitochondrial fission marker FIS1 and mitophagosome abundances were increased but mitochondrial fusion marker OPA1 was decreased in NG-HTN compared to Control hearts (Figures 7B,C). This was corroborated by, our *in vitro* data with H9c2 cardiomyocytes treated with NE showing a decrease in mitochondrial biogenesis (Figure 7D). Overall, these data support the general hypothesis that mitochondrial biogenesis, dynamics, and mitophagy are suppressed with enhanced neurogenic tone in NG-HTN hearts.

### Mitochondrial Proteinopathies

**FIGURE 8:**
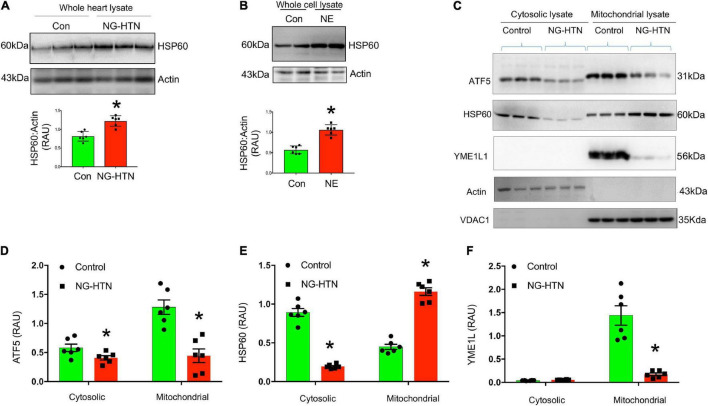
Neurogenic hypertension (NG-HTN) downregulates mitochondrial UPR (UPR^mt^). **(A)** Representative Western blots and quantification of cardiac HSP60 expression normalized to Actin in control and NG-HTN whole hearts lysate. **(B)** Representative Western blots and quantification of HSP60 expression normalized to Actin in NE-treated H9c2 whole cell lysate. **(C)** Representative Western blots and **(D–F)** quantifications of cytosol vs. mitochondrial ATF5, HSP60, and YME1L1 expression level normalized to loadings controls in NG-HTN and control hearts. Values are mean ± SEM, dot represents *n* = 6 rats, *P < 0.05 vs. Control, **(A,B)**, Student’s *t*-test, **(D–F)**; two-way ANOVA with Tukey’s multiple comparison test.

To determine if NG-HTN causes changes in mitochondrial protein import, TIM and TOM translocase, key sub-units involved in mitochondrial protein import were measured. Levels of TOM20 are decreased in NG-HTN, while levels of TIM17 are increased (Figures 7E,F), which indicates an altered mitochondrial protein import efficiency and YME1L1 protease activation (Rainbolt et al., 2013; Figures 8C,F) and thus associated with the overall compromised mitochondrial proteostasis in the hearts of rats with NG-HTN.

### Mitochondrial Proteotoxic Stress

To determine if NG-HTN causes mitochondrial proteotoxic stress and downregulates UPR^mt^ we measured levels of ATF5, a hallmark UPR^mt^ marker (Figures 8C,D). In the absence of mitochondrial stress, ATF5 is targeted to mitochondria and accumulates within mitochondria. However, during mitochondrial respiratory chain dysfunction, high levels of ROS, and during mitochondrial protein folding stress, ATF5 fails to import into mitochondria and a percentage of ATF5 accumulates in the cytosol and traffics to the nucleus where it activates the UPR^mt^ that promote cascade of gene activation for mitochondrial proteostasis and the recovery of depolarized/defective mitochondria. Our results suggest that ATF5 protein level is reduced in both, cytosol and in mitochondria of NG-HTN hearts compared to control hearts, suggesting an UPR^mt^ downregulation in NG-HTN (Figures 8C,D). Since ATF5 increased HSP60 therefore we measured and compared HSP60 protein level in cytosol, mitochondria, as well as in whole heart tissue lysates (Figure 8A). The heat shock protein 60 (HSP60, is another UPR^mt^ marker downstream of ATF5), which is a chaperon responsible for refolding and transportation of nuclear-encoded mitochondrial proteins from the cytoplasm into the mitochondrial matrix under stress conditions. There was increased UPR^mt^ activation marker HSP60, in the NG-HTN group whole heart tissue lysate indicating an altered mitochondrial proteotoxic stress in response to mitochondrial abnormality (Figure 8A). However, further analyses of HSP60 in cytosol to mitochondria showed that HSP60 protein level is decreased in cytosol but increased in mitochondrial compartments of NG-HTN (Figures 8C,E). Furthermore, our *in vitro* data using H9c2 cardiomyocytes treated with NE showed congruent result of increased HSP60 in whole cell lysate (Figure 8B). To understand a mechanistic link to these observations, we studied the mitochondrial AAA (ATPases associated) YME1L1 protease that involved in the quality control and processing of mitochondrial inner-membrane proteins. Reports suggests that YME1L1 (a mitochondrial AAA protease) controls the accumulation of respiratory chain supercomplex subunits and depletion of YME1L1 led to excessive accumulation of non-assembled respiratory chain subunits (Ndufb6, ND1, and Cox4) in the inner membrane (Stiburek et al., 2012; Rainbolt et al., 2013). Therefore, we investigated the activity of YME1L1 in the mitochondrial compartment of NG-HTN hearts and control hearts. Our results demonstrate the YME1L1 is highly suppressed in the mitochondria of NG-HTN (Figures 8C,F), supporting the hypothesis of non-assembled/misfolded mitochondrial inner membrane OXPHOS proteins accumulation (Figure 6B). All these results suggests that a decreased ATF5 import, and lack of UPR^mt^ activation increased OXHOS assembly defects in the mitochondria of NG-HTN hearts. Overall, our data support the notion that an exaggerated neurogenic drive causes mitochondrial proteotoxic stress.

### Neurogenic Hypertension Reduces MiR-18a-5p but Increases Cardiac HIF-1α

**FIGURE 9:**
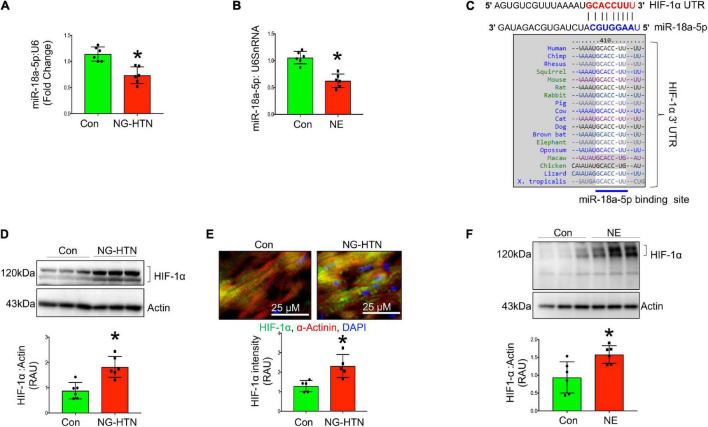
Neurogenic hypertension (NG-HTN) and miR-18a-5p/HIF-1α axis. **(A)** Quantification of miR-18a-5p level in control and NG-HTN hearts normalized to U6SnRNA. **(B)** Quantification of miR-18a-5p level in control and NE-treated H9c2 cardiomyocytes normalized to U6SnRNA. **(C)** *In silico* prediction of miR-18a-5p binding on 3′UTR of HIF-1α and its species conserveness. **(D)** Western blot and quantification showing expression of HIF-1α in control and NG-HTN rats normalized to Actin. **(E)** Immunofluorescence assay and quantification showing HIF-1α localization in control and NG-HTN hearts (HIF-1α, green; α-actinin, red; and DAPI, blue). **(F)** Western blot and quantification showing expression of HIF-1α normalized to Actin in control and H9c2 cardiomyocytes treated with NE. Values are mean ± SEM, dot represents *n* = 6 rats, *P < 0.05 vs. Control, Student’s *t*-test.

**FIGURE 10:**
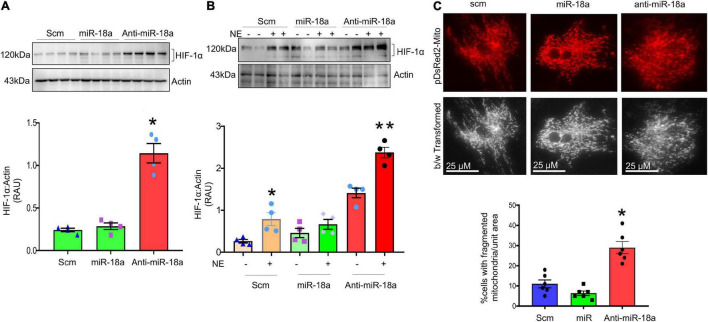
MiR-18a-5p targets HIF-1α and mitochondrial fission. **(A)** Western blot and quantification showing expression of HIF-1α normalized to Actin in the H9c2 cardiomyocytes treated with scm, miR-18a-5p, and anti-miR-18a-5p. **(B)** Western blot and quantification showing expression of HIF-1α normalized to Actin in H9c2 cardiomyocytes treated by scm, miR-18a-5p, and anti-miR-18a-5p, with NE or no NE. Values are mean ± SEM, *P < 0.05 vs. scm, **P < 0.05 vs. miR-18a-5p. One-way ANOVA. **(C)** Top, live cell pDsRed2-Mito image of H9c2 cardiomyocytes treated with scm, miR-18a-5p, and anti-miR-18a-5p. Bottom, quantification of % number of cells with fragmented mitochondria (all globular) per unit areas in each group from six independent experiments.

Levels of miR-18a-5p are significantly decreased in the hearts of rats with NG-HTN (Figure 9A). Further, our *in silico* data predicted that miR-18a-5p targets HIF-1α 3′UTR mRNA (Figure 9C). HIF-1α protein expression was evaluated using Western blotting of left ventricular cardiac tissues of Control and NG-HTN groups. HIF-1α protein levels are increased in the NG-HTN compared to Control rats (Figure 9D). Further, immunofluorescence of HIF-1α demonstrated a distinct increase in cellular and nuclear immunolabeling of HIF-1α in cardiomyocytes of rats with NG-HTN (Figure 9E). Consistent with these data, direct treatment of NE on H9c2 cardiomyocytes *in vitro*, demonstrated reduced miR-18a-5p level with a concomitant increase in the expression of HIF-1α as well as cardiomyocyte hypertrophy (Figures 5A–E, 9B,F). To further examine the relationship between miR-18a-5p and HIF-1α we transfected H9c2 cardiomyocytes with miR-18a-5p or anti-miR-18a-5p mimic probes to elicit either miRNA gain or loss of function, respectively, and then examined HIF-1α protein levels after 24 h treatment. In addition, we transfected cardiomyocytes with scrambled (scm) miRNA as a negative “control mimic.” Loss of miR-18a-5p significantly increased HIF-1α protein levels in cardiomyocytes (Figure 10A), which indicates that miR-18a-5p inhibits the expression of HIF-1α, however in normal or unstressed cells miR-18a-5p mimic forced overexpression maintains basal HIF-1α protein level by finetuning mRNA stability and protein translation. Notably, the gain of function by miR-18a-5p overexpression efficiently blunted NE-induced HIF-1α increase in H9c2 cardiomyocytes (Figure 10B).

To further investigate whether, increased HIF-1α contributes to a change in mitochondrial dynamics in cardiomyocytes we monitored the mitochondrial network using pDsRed2-Mito plasmid transfection in the cardiomyocytes, a mitochondria-reporter clone that visualizes mitochondria network with red fluorescence. The pDsRed2-Mito vector transfection visualized tubular shaped mitochondrial structures to identify fused mitochondria and globular shaped structures representing fragmented mitochondria. Our results demonstrated that anti-miR-18a-5p treatment increased fragmented or globular mitochondrial structures indicating increased mitochondrial fission (Figure 10C).

### Link Between MiR-18a-5p and HIF-1α

**FIGURE 11:**
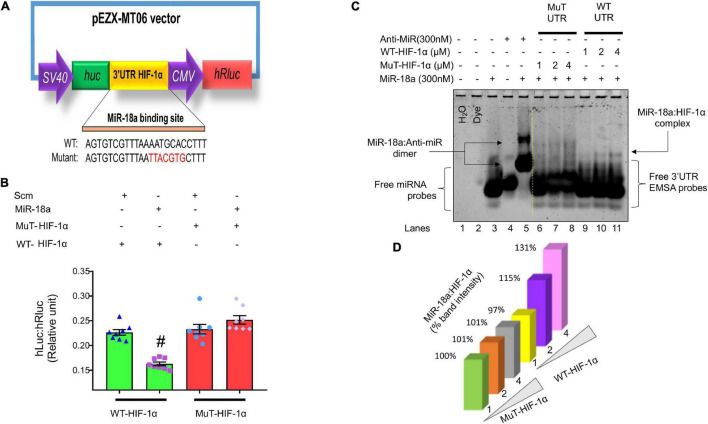
MiR-18a-5p targets HIF-1α 3′UTR. **(A)** Dual-luciferase reporter assay construct showing WT and mutant miR-18a-5p binding sequences. **(B)** Luciferase reporter quantification showing relative expression of luciferase activity in miR-18a-5p, or scrambled miRNA-treated H9c2 cells co-transfected with either WT or mutant HIF-1α 3′UTR, dot represents individual values, *n* = 8 wells/group. ^#^*P* < 0.05 vs. WT-HIF-1α 3′UTR, two-way ANOVA. **(C)** MiRNA–mRNA EMSA showing binding affinity of miR-18a-5p with WT or mutant HIF-1α 3′UTR (1, 2, or 4 μM). **(D)** Quantification of miR-18a-5p % band intensity shift with 1, 2, or 4 μM of WT or mutant HIF-1α 3′UTR promoter.

*In silico* analysis indicates that HIF-1α is a potential target for miR-18a-5p (Figure 9C). To determine whether miR-18a-5p targets HIF-1α 3′ untranslated region (3′UTR), we performed luciferase reporter assay using WT 3′UTR and mutant 3′UTR clones of HIF-1α (Figures 11A,B). In Mut HIF-1α 3′UTR clone the miR-18a-5p binding seed sequence region on 3′UTR of HIF-1α was mutated. The H9c2 rat cardiomyocytes were transfected with either with WT HIF-1α 3′UTR or Mut HIF-1α 3′UTR luciferase assay clones, with miR-18a-5p or scrambled miRNAs overexpression and then the relative firefly to Renilla luciferase activities was assessed. MiR-18a-5p decreased relative firefly to Renilla luciferase activity in WT HIF-1α 3′UTR compared to scrambled, suggesting that miR-18a-5p targets WT HIF-1α 3′UTR. As anticipated, luciferase activity remained unchanged with miR-18a-5p mimic treatment to Mut HIF-1α 3′UTR (Figure 11B), which confirms that miR-18a-5p targets HIF-1α 3′UTR.

**FIGURE 12:**
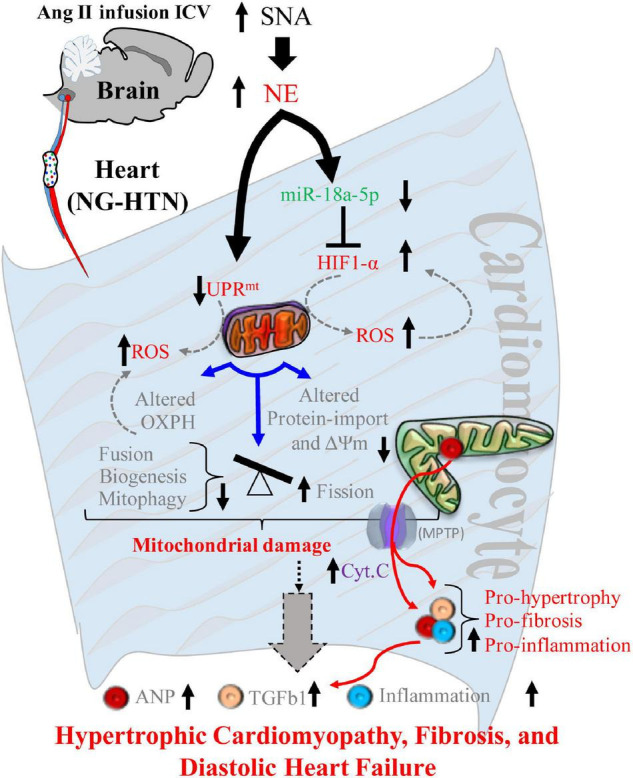
Schematic showing mechanisms of mitochondrial dysfunction in central Ang II (ICV) infusion induced NG-HTN. Schematic model showing proposed molecular mechanisms causing mitochondrial abnormality in the heart of rat with neurogenic hypertension resulting in progression to hypertrophic cardiomyopathy. An increased sympathoexcitation (SNA) induces an increase in cardio-neuronal norepinephrine which causes either UPR^mt^ and/or miR-18a-5p/HIF-1α axis dysregulation that leads to increased mitochondrial ROS generation. Increased ROS alters mitochondrial OXPHOS, protein-import, and mitochondrial dynamics to tip the balance of Fusion/Fission to greater Fission, leading to overall mitochondrial abnormality. These underlies ROS induced release of mitochondrial cytochrome-c into cytoplasm *via* mitochondrial permeability transition pores (MPTP) opening eventually mitochondrial abnormality that progressively contributes to hypertrophic remodeling, fibrosis, inflammation and diastolic heart failure in the rats with NG-HTN.

To further confirm that miR-18a-5p target HIF-1α 3′UTR seed sequence, we performed miRNA–mRNA electrophoretic mobility shift assay (miRNA–mRNA EMSA; Nandi and Mishra, 2017) using WT HIF-1α 3′UTR and Mutant HIF-1α 3′UTR EMSA oligonucleotide probes in the presence and absence of miR-18a-5p or anti-miR-18a-5p mimic probes (Table 2). In miRNA-EMSA, the miR-18a-5p and anti-miR-18a-5p display one band (Figure 11C, lanes 3 and 4). The incubation of miR-18a-5p with an equimolar (300 nM) concentration of anti-miR-18a-5p reduces the mobility of miR-18a-5p band due to the formation of miR-anti-miR dimer complex (Figure 11C, lane 5). Furthermore, the miR-18a-5p probe incubation with three different concentrations (1, 2, and 4 μM) of WT HIF-1α 3′UTR form a low mobility second band of miR-18a-WT HIF-1α 3′UTR complex (Figure 11C, lanes 9, 10, and 11). This second band represents the binding of the miR-18a-5p with WT HIF-1α 3′UTR seed binding sequence. Noticeably, there was an increased band intensity of this second band with increasing concentrations of WT HIF-1α EMSA probe (Figures 11C,D, lanes 9, 10, and 11). To further verify the specificity of miR-18a-5p-WT HIF-1α 3′UTR complex, we used respective concentrations (1, 2, and 4 μM) of Mut HIF-1α 3′UTR EMSA probes, which had mismatch mutations with the miR-18a-5p seed sequence binding region. As expected, we did not observe a low mobility second band formation with Mut HIF-1α 3′UTR probes suggesting an absence of miR-18a-5p-Mut-HIF-1α 3′UTR complex formation (Figure 11C, lanes 6, 7, and 8). Altogether, our target validation data demonstrated the specificity of miR-18a-5p binding to WT HIF-1α 3′UTR, which confirms that miR-18a-5p targets HIF-1α and mitochondrial function (Figures 11, 12).

**TABLE 2:** Details of PCR primers and EMSA probes.

Probe name	Oligonucleotide sequences
D-Loop F	5′-CTACCATCCTCCGTGAAAC-3′
D-Loop R	5′-TGATTAGACCCGATACCATC-3′
gGAPDH F	5′-AAGCGGACTTACAGAGGTC-3′
gGAPDH R	5′-ACTACAGGAGCCATTTTGTG-3′
Wild type HIF-1α 3′UTR EMSA	5′-ATCATTTTAAAAAATGCACCTTT-3′
Mutant HIF-1α 3′UTR EMSA	5′-ATCATTTTAAAAAATTCAGCCTA-3′
MiR-18a-5p EMSA	5′-TAAGGTGCATCTAGTGCAGATAG-3′
Anti-miR-18a-5p EMSA	5′-CTATCTGCACTAGATGCACCTTA-3′

## Discussion

There is a strong link between cardiac pathogenesis and changes in mitochondrial quality and function. However, mitochondrial changes in the progression of cardiac diseases is not yet fully understood, particularly little is known as to how mitochondrial dysfunction is initiated in cardiac disease to cause final cardiac pathology. The present study shows that central Ang II infusion causes enhanced sympatho-excitation that leads to hypertension in rats. In response to sympatho-excitation, these hypertensive rats demonstrate pathological cardiac hypertrophy and fibrosis, with concomitant abnormal mitochondrial function due to multiple events, such as altered mitochondrial ultrastructure, compromised OXPHOS, impaired structural dynamics and biogenesis, suppressed UPR^mt^ and protein quality control that is associated with reduced proteostasis, altered pre-protein transport activities, impaired mitophagosome clearance and increased ROS generation. At the same time there was decreased miR-18a-5p expression (a cardiac anti-hypertrophic, anti-fibrotic) and increased HIF-1α level (a glycolytic activator that suppress mitochondrial metabolism and function) in the hearts of rat with NG-HTN. Further, we observed an inverse relationship between miR-18a-5p expression and HIF-1α levels. Finally, we demonstrated that norepinephrine directly down-regulates miR-18a-5p expression. Taken together, these data demonstrates that enhanced sympathetic tone initiates a reduction in miR-18a-5p expression, a potential HIF-1α targeting miRNA which in turn allows for an increase in HIF-1α levels that leads to altered cardiac metabolic shift and increased ROS generation mediated mitochondrial dysfunction. These results are consistent with cardiac mitochondrial complications that are reported during hypertension, such as increased cellular/mitochondrial ROS, compromised mitochondrial OXPHOS, and mitochondrial abnormality (Eirin et al., 2014; Power et al., 2016). It is postulated that persistence cardio-neuronal norepinephrine overstimulation in the heart of rat with NG-HTN activates cytosolic and mitochondrial Ca_2_^+^ overload that results in increased Calcium–Calcineurin–NFAT, and enhanced noradrenergic tone induced desensitization of β-AR signaling *via* receptor internalization (Almela et al., 2020; Boyman et al., 2021). These changes need an adaptive shift in mitochondrial energy fueling and QC changes to accommodate the hypertensive cardiac contractility, requiring increased mitochondrial workload *via* alternate substrate utilization. This causes leaky mitochondrial respiration and increase mitochondrial ROS generation. The increased mitochondrial ROS generation opens mitochondrial transition pores (MPTP; Javadov et al., 2009) to release mitochondrial cytochromes into cytoplasm and to activate pro-hypertrophy/fibrosis/apoptotic and necrotic factors. Eventually this causes pathological cardiac hypertrophy, fibrosis and associated secondary inflammation from the circulation in the heart in response to sympatho-excitation commonly observed in NG-HTN that progress to heart failure over the time.

Some of the previous evidence for this were obtained while studying animal model of hypertension induced by subcutaneous delivery of Ang II, where the effects of Ang II is on the peripheral circulation including the heart to increase the blood pressure (Dai et al., 2011; Wei et al., 2017) and not specifically directed to the central nervous system. This model of hypertension initially promotes a compensatory cardiac hypertrophy due to peripheral vasoconstriction-mediated increased afterload, which later progresses to hypertension mediated heart failure (Dai et al., 2011; Power et al., 2016). However, whether these cardiac mitochondrial impairments are initiated by the enhanced sympathetic drive to the heart (*per se*) leading to NE programmed left ventricular remodeling in hypertension remains unclear (Zucker et al., 2001; Zhu et al., 2002). The hallmarks of hypertensive cardiomyopathy are hypertrophy, fibrosis, ECM expansion, apoptosis, inflammation, impaired autophagy/mitophagy, and vascular complications (Wang et al., 2010; Eirin et al., 2014). Our current model of hypertension is unique in that it purports to elicit a neurogenic cause for the cardiac excitation with concomitant elevated vascular resistance leading to the development of hypertension and consequent cardiomyopathy progression. Therefore, we studied the development of heart disease in this neurogenic model of hypertension. Our results confirmed that NG-HTN increased cardiac hypertrophy and fibrosis, and diastolic dysfunctions in the hearts of these rats, associated with increased general sympathetic activation.

The nuclear encoded mitochondrial protein quality controls are maintained by several signaling events both in the cytosol and mitochondria (Schmidt et al., 2010), which remain unstudied in the NG-HTN-induced cardiomyopathies. Mitochondrial dysfunction induces retrograde signaling of UPR^mt^ activation for mitochondrial proteostasis (Melber and Haynes, 2018). Activation of UPR^mt^ by mislocalized/misfolded mitochondrial precursor (pre) proteins indicates a compromised cytosol to mitochondrial protein import (Wrobel et al., 2015). Here, we determined the impact of elevated sympatho-excitation on mitochondrial UPR^mt^ downregulation and increased mitochondrial proteotoxic stress signaling for the progression of NG-HTN-induced cardiomyopathy. We further identified a direct link between NE in mitochondrial UPR^mt^ and proteotoxic stress. Since mitochondrial dysfunction exhibits increased ROS generation and compromised OXPHOS, therefore, we focused our studies on measuring the effect of enhanced neurogenic sympathetic tone on mitochondrial ROS generation and OXPHOS event. Our data demonstrated that NG-HTN increased mitochondrial ROS levels and altered expression of mitochondrial respiratory complexes. Consistent with these observations, the direct application of NE *in vitro* on H9c2 cardiomyocytes showed increased mitochondrial ROS levels and decreased mitochondrial membrane potential suggesting that noradrenergic activation cascade initiated these changes in the mitochondria. In addition to this, our data uncovered an abundant amount of abnormal mitochondria in myocardial cells under EM, suggesting structurally impaired and functionally compromised mitochondria in the hearts of rats with NG-HTN, which is analogous to what is observed in clinical hypertension and related heart disease (Dikalov and Dikalova, 2016; Wei et al., 2017). Our results are also congruent with the previous reports which demonstrated that mitochondrial abnormalities are one common feature in all types of cardiomyopathies and increased mitochondrial ROS compromised mitochondrial respiration (Power et al., 2016). Based on these observations one may speculate that perhaps the neurogenic component may be the critical factor for these observed alterations in all forms of hypertension.

The mitochondrial network in cardiomyocytes undergoes biogenesis, fission/fusion, and mitophagy events to maintain a balance of healthy pool of mitochondria. Fragmented mitochondrial phenotypes, lack of fusion, and decreased biogenesis has been observed during diverse pathological cardiac remodeling and heart failure (Chen et al., 2009; Ong et al., 2010). However, the mechanisms which underly the changes in mitochondrial dynamics during hypertrophic growth of cardiomyocytes is not entirely clear. Our data demonstrate that mitochondrial biogenesis is decreased in the hearts of rats with NG-HTN, suggesting a dysfunction in mitochondrial biogenesis that does not manage to recover damaged mitochondrial loss *via* mitophagy. Our results further demonstrated that mitochondrial fission is increased and fusion is decreased in the heart of rat with NG-HTN, which are in accordance with the data presented with FIS1 and OPA1 that further corroborates with increased fission and decreased fusion events of the mitochondria in the heart of NG-HTN (Papanicolaou et al., 2011; Ikeda et al., 2015). Further, EM data demonstrated attenuated mitochondrial area coupled with decreased cristae area, implicating compromised mitochondrial function including OXPHOS. This is supported by the presence of meAPs as well as increased numbers of miAPs, indicating impairment of mitophagy events. However, whether mitophagy flux is decreased or increased in hypertensive heart disease remains to be determined definitively using mitophagy reporter animal models. Our findings are consistent with previous reports in hypertensive hearts showing an abundant amount of abnormal mitochondria in myocardial cells examined by EM, which support impaired mitophagy (Wei et al., 2017; Nandi et al., 2020a). Decreased mitochondrial biogenesis, fission-fusion dynamics, and mitophagy leading to mitochondrial abnormality in NG-HTN was not explored prior to this study. In this regard, NE signaling has been purported to play a significant role in cardiac remodeling and hypertrophy, adaptations (Barki-Harrington et al., 2004). It is of interest to note that NE has been reported to increase mitochondrial fission and suppress mitochondrial function in the heart (Pennanen et al., 2014). Furthermore, cardiomyocyte hypertrophy has been associated with diminished mitochondrial metabolism in compensatory hypertrophied myocardium of hypertensive rats (Power et al., 2016). Other reports suggest that mitochondrial fusion events *via* OPA1 or MFN1/2 appear to decrease in heart failure (Javadov et al., 2011; Papanicolaou et al., 2011), as well. In accordance with these reports, our results demonstrated that treatment of cultured H9c2 rat cardiomyocytes with NE increased cardiomyocyte hypertrophy, decreased mitochondrial biogenesis, ΔΨm, and increased mitochondrial fission and ROS generation and shifted OXPHOS. Therefore, these data strongly suggest an initiating role for enhanced neurogenic tone as a possible origin for the impaired mitochondrial dynamics. The overall assessments of norepinephrine effect on heart and cardiomyocytes showed changes in mitochondrial quality control parameters indicating reduced mitochondrial functional capacity. These data further suggest a potential role of NE→reduced UPR^mt^→ROS and miR-18a-5p/HIF-1α→ROS induced decrease in mitochondrial fission-fusion dynamics and biogenesis in the development of sympatho-excitation-induced cardiac hypertrophic remodeling. Notably, these changes are accomplished *via* OXPHOS metabolic shifts toward glycolytic substrates utilization for cardiac hypertrophy (Dai et al., 2011; Chu et al., 2012). In this context, cardiomyocyte hypertrophy has been reported with diminished mitochondrial metabolism, and fragmentation of the mitochondrial network precedes metabolic alterations in response to norepinephrine (Pennanen et al., 2014). Furthermore, our results showed that lack of miR-18a-5p increased mitochondrial fragmentation in hypertrophic cardiomyocyte in response to norepinephrine. Related studies demonstrated that inhibiting mitochondrial fission protects the heart against ischemia/reperfusion injury (Ong et al., 2010) and mdivi-1, a pharmacological inhibitor for mitochondrial fission inducer Drp1, ameliorated pressure overload-induced heart failure in mice (Givvimani et al., 2012). In addition, a dominant negative Drp1, is reported to blunt hypertrophic growth, and prevent norepinephrine-induced reduced mitochondrial function (Pennanen et al., 2014). Moreover, a deficiency of mitochondrial fusion inducer Mfn2 further showed cardiac hypertrophy and reduced cardiac function (Papanicolaou et al., 2011). Taken together, all these reports support the notion that mitochondrial dynamics play a crucial role in the development of heart diseases in the downstream of sympathetic activation. Therefore, our results imply that targeting mitochondrial dynamics can be a potential therapeutic approach for preserving mitochondrial function for cardioprotection in NG-HTN.

In addition to UPR^mt^ marker ATF5 downregulations there was a concomitant decreased expression of mitochondrial outer membrane translocase proteins TOM20 but increased abundance of nuclear-encoded mitochondrial inner membrane protein TIM17A in NG-HTN hearts (Rainbolt et al., 2013). These results suggest that the nucleus to mitochondria communication for mitochondrial pre-protein import and proteostasis is reduced in the NG-HTN myocardium. Our results suggest a notion that in NG-HTN hearts several nuclear encoded mitochondrial pre-proteins transcription or translation are suppressed due to suppressed UPR^mt^ stress response signal inside the mitochondrial matrix. Our cytosol vs. mitochondrial western blot data demonstrated that in control hearts the key UPR^mt^ marker ATF5 is imported into mitochondria. However, due to mitochondrial proteotoxic stress in NG-HTN, ATF5 fails to be imported into mitochondria, and cytosolic ATF5 level is also reduced in NG-HTN, indicating UPR^mt^ downregulation. In addition, transcriptional factor ATF5, which regulates expression of key downstream UPR^mt^ genes like HSP60 showed its increased protein level of accumulation in the NG-HTN mitochondria, while HSP60 cytosolic level is reduced in NG-HTN. Furthermore, assessment of the mitochondrial quality-control protease YME1L1, a central to UPR^mt^ signal transduction for OXPHOS subunit assembly/import, was reduced in the mitochondrial matrix of NG-HTN. These changes suppressed UPR^mt^ and perhaps increases the OXPHOS subunits misfolded supercomplex accumulation, and increased HSP60 level in mitochondrial fraction with a decreased Mn-SOD antioxidant capacity as we noticed with our Western blots. The turnover of HSP60 stabilization in NG-HTN mitochondria may possibly increases to cope the newly synthesized mitochondrial genome encoded proteins folding overload and their subsequent assembly. We therefore concluded that ATF5 and HSP60 signal mechanisms are complex and perhaps regulated by different axes of the canonical or non-canonical UPR^mt^ in NG-HTN heart. Notably, UPR^mt^ activation eliminates severely defective or damaged mitochondria *via* mitophagy, however a suppressed UPR^mt^ in NG-HTN heart perhaps increased mega-autophagosome accumulation as we noticed with EM evaluations (Pellegrino et al., 2013). In addition, the decreased mtDNA content in NG-HTN indicates a reduced new mitochondrial biogenesis, which further supports a lack of UPR^mt^ activation in the heart of NG-HTN (Shpilka et al., 2021). Furthermore, our data suggested that a weaker ATF5 signal in NG-HTN hearts may allow a reduction signal for mitochondrial overall protein import capacity. However, the UPR^mt^ is highly complex and how the UPR^mt^ is regulated in mammal still remains unclear (Fiorese et al., 2016). Therefore, further study is necessary to understand the signaling communications from the cytosol to mitochondria, and nucleus to mitochondria for the mitochondrial proteostasis in NG-HTN heart and if mitochondrial matrix proteases preferentially regulates OXPHOS complexes to stablish an ETC or metabolic switch due to mitochondrial stress and reduced the pre-protein import that leading to the accumulation of unassembled subunits of ETC complexes or causing canonical or non-canonical UPR^mt^ axes are unclear at present (Nargund et al., 2012; Melber and Haynes, 2018; Rolland et al., 2019).

Several reports suggest that mitochondrial dysfunction presents a new horizon to predict the progression of hypertension cardiomyopathy (Drazner, 2011; Nadruz, 2015; Eirin et al., 2018). Considering that one-third of the intracellular volume of a cardiomyocyte is mitochondria, our results support the general hypothesis that hypertension-induced mitochondrial abnormality in cardiomyocytes is a major contributor to NG-HTN-induced cardiac hypertrophy and fibrotic remodeling. Further, our *in vitro* data with direct action of NE on cultured cardiomyocytes corroborates the concept that enhanced sympathetic tone is the initiating factor for these mitochondrial injuries leading to hypertensive cardiomyopathy. In this regard, it has been well documented that mitochondrial abnormality is associated with hypertensive heart diseases (Eirin et al., 2014, 2018; Dikalov and Dikalova, 2016). However, the mitochondrial impairments that prevails in NE-induced programmed left ventricular remodeling associated with hypertension remained unclear until the current study.

Several miRNAs have been reported to be lower during hypertension (Lee et al., 2015; Seeger and Boon, 2016). The cause for this decrease is not entirely clear. The results from this study demonstrate that concomitant with the sympatho-excitatory state in NG-HTN there is decreased levels of miR-18a-5p. Furthermore, our *in vitro* data using cardiomyocyte cultures clearly demonstrated that direct application of NE causes a decrease in miR-18a-5p. Taken together these data provides a possible mechanistic link between sympatho-excitation and reduced levels of cardiac miR-18a-5p in NG-HTN (Huang et al., 2017; Nandi et al., 2020b).

Previously we reported that downregulation of anti-hypertrophic and anti-fibrosis miRNAs can induce cardiac fibrosis and hypertrophy (Nandi et al., 2016). MiRNAs have been reported to lower hypertension in SHR by upregulating mitochondrial translation (Li et al., 2016). MiR-18a-5p has been purported to be an anti-hypertrophy/anti-fibrosis miRNA in the heart (Huang et al., 2017). On the other hand, persistent activation of HIF-1α promotes cardiac hypertrophy in hypertension (Kumar et al., 2018) and increased HIF-1α suppresses mitochondrial function (Kim et al., 2006; Papandreou et al., 2006). Considering these facts together we endeavored to explore whether there was a link between miR-18a-5p and HIF-1α in NG-HTN. Our *in silico* analysis predicted that miR-18a-5p targets HIF-1α. Our data demonstrated that when miR-18a-5p is downregulated the levels of HIF-1α protein is increased in the hearts of rats with NG-HTN, concomitantly. Moreover, increased HIF-1α is associated with hypertrophy in the hearts and cultured neonatal rat cardiac myocytes, which is consistent with the role of HIF-1α in the heart for hypertension-induced hypertrophic cardiac remodeling *via* mitochondrial suppression (Kumar et al., 2019). HIF-1α promotes glycolytic activation and suppresses mitochondrial function (Kim et al., 2006; Papandreou et al., 2006). In addition, our *in vitro* data using cardiomyocyte cultures clearly demonstrated that direct application of NE causes a decrease in miR-18a-5p with a concomitant increase in HIF-1α and induced molecular markers of cardiomyocyte hypertrophy and mitochondrial dysfunction. These results demonstrate an inverse relationship between levels of miR-18a-5p and levels of HIF-1α suggesting that conceivably miR-18a-5p inhibits HIF-1α.

These result supports the overall hypothesis that NE reduces the levels of miR-18a-5p which in turn allows for an increase in the levels of HIF-1α which in turn involved in suppressed mitochondrial function in cardiac myocytes. Our data show that NG-HTN reduces miR-18a-5p levels and at the same time increases cardiac HIF-1α. In addition, we have shown that miR-18a-5p targets HIF-1α 3′UTR and binds to HIF-1α 3′UTR seed sequence to regulate its function using miRNA 3′URT dual-luciferase assay and miRNA–mRNA EMSA assays as well. Furthermore, miR-18a-5p overexpression blunted norepinephrine-induced HIF-1α increase in cardiomyocytes, and lack of miR-18a-5p in *in vitro* H9c2 cardiomyocytes increased mitochondrial fragmentation. These results further suggesting that the levels of miR-18a-5p regulate levels of HIF-1α and thus hypertrophic cardiac remodeling *via* mitochondrial suppression (Kumar et al., 2019).

In conclusion, we have demonstrated that chronic Ang II (ICV) infusion induces exaggerated sympatho-excitation mediated NG-HTN in rats. Our study has identified a critical neural link operating *via* ROS, metabolism, and miRNA signaling pathway that produces mitochondrial stress leading to cardiac hypertrophy, fibrosis, and progression to cardiomyopathy/heart failure in our rat model of NG-HTN. The relationship between mitochondrial dynamics and mitochondrial stress in hypertensive cardiac hypertrophy is presented in the study. A stimulation of the adrenergic receptors by enhanced noradrenergic tone perhaps leads to an increase in mitochondrial calcium and OXPHOS overload and associated mitochondrial UPR^mt^ dysfunctions. Our results identify a new potential therapeutic target for the prevention of pathological hypertensive hypertrophy *via* targeting mitochondria. We showed that norepinephrine promotes mitochondrial fission *via* mitochondrial ROS, proteotoxicity, dynamics alteration, and by shifted metabolism. Possibly, a balance between mitochondrial fission, fusion, biogenesis, mitophagy, and altered NE→reduced UPR^mt^→ROS and miR-18a-5p/HIF-1α→ROS axis may be sufficient to prevent cardiomyocyte hypertrophy and hypertension cardiomyopathy. The understanding of these fundamental molecular mechanisms provides us critical insight into the possible development of novel therapeutics for alleviating the cardiac abnormality commonly observed in hypertensive heart disease in the future.

## Limitations

1. The results shown with this NG-HTN model are unique and novel since they delineate the effect of symptho-excitation specifically on cardiac mitochondrial function and cardiac remodeling. Although clinically all hypertensives patients have some neural contribution, there are potentially other factors that may also play a role.
2. It could be argued that sympatho-excitation may cause an increase in peripheral Ang II which in turn may induce the production of mitochondrial reactive oxygen species. Nevertheless, it is safe to assume that direct effects of cardio-neuronal mechanisms are primarily responsible for cardiac remodeling in the current model. We studied the NG-HTN induced changes with 14 days of Ang II infusion, however, an infusion study over longer period of time remains to be examined.
3. Contribution of vascular constriction, dysfunction, and/or a systemic inflammation mechanism in response to NG-HTN may contribute to the changes in cardiac abnormalities. These factors remain to examined.
4. We proposed an impairment of mitochondrial OXPHOS shift in the heart of rat with NG-HTN, which provokes cardiac remodeling. However, it is acknowledged that this mechanism is not directly tested in this study.
5. Our *in vitro* results validated that miR-18a-5p functionally targets HIF-1α and is directly involved in regulating mitochondrial structures. However, the contribution of miR-18a-5p/HIF-1α axis relevance to mitochondrial UPR *in vivo* remains to be examined in gain or loss of function models.

## Future Perspective

Mitochondrial abnormalities is commonly reported in the hearts of patients with hypertension. However, the source of what elicits this cardiac mitochondrial impairments leading to heart failure remains to be explored. Present study demonstrated that neurogenic hypertension reduced cardiac miRNA-18a-5p and increased HIF-1α, with concomitant mitochondrial proteinopathy, UPR^mt^ stress, mitochondrial structural abnormalities, increased mitochondrial ROS, altered oxidative phosphorylation and reduced mitochondrial biogenesis and fusion, but increased fission. This study suggests that chronic sympathoexcitation to the heart contributes to pathological cardiac remodeling *via* NE→miR-18a-5p/HIF-1α axis and UPR^mt^ to alter mitochondrial function. Targeting this pathway therapeutically would provide a novel approach.

## Data Availability Statement

The raw data supporting the conclusions of this article will be made available by the authors, without undue reservation.

## Ethics Statement

The animal study was reviewed and approved by all experimental protocols were approved by the Institutional Animal Care and Use Committee, University of Nebraska Medical Center, and all protocols/methods were conducted in accordance with the relevant guidelines and regulations of our institution, the American Physiological Society, and the National Institutes of Health Guide for the Care and Use of Laboratory Animals.

## Author Contributions

SN conceived, designed, and co-ordinated the research plans, performed surgeries, generated and interpreted the data, wrote and edited the manuscript. SN and KK contributed to the hemodynamics analyses. SM performed EM studies. KP supervised all aspects of the study and the manuscript. All authors contributed to the article and approved the submitted version.

## Conflict of Interest

The authors declare that the research was conducted in the absence of any commercial or financial relationships that could be construed as a potential conflict of interest.

## Publisher’s Note

All claims expressed in this article are solely those of the authors and do not necessarily represent those of their affiliated organizations, or those of the publisher, the editors and the reviewers. Any product that may be evaluated in this article, or claim that may be made by its manufacturer, is not guaranteed or endorsed by the publisher.
